# The color communication game

**DOI:** 10.1038/s41598-023-42834-3

**Published:** 2023-09-25

**Authors:** Angela M. Brown, Delwin T. Lindsey

**Affiliations:** 1 Ohio State University College of Optometry, 338 West 10th Ave, Columbus, OH 43210-1280 USA; 2 Department of Psychology, Ohio State University, Ovalwood Hall, 1680 University Drive, Mansfield, OH 44906-1547 USA

**Keywords:** Human behaviour, Computational science

## Abstract

There is clear diversity among speakers of a typical language in how colors are named. What is the impact of this diversity on the people’s ability to communicate about color? Is there a gap between a person’s general understanding of the color terms in their native language and how they understand a particular term that denotes a particular color sample? Seventy English-speaking dyads and 63 Somali-speaking dyads played the Color Communication Game, where the “sender” in each dyad named 30 color samples as they would in any color-naming study, then the “receiver” chose the sample they thought the sender intended to communicate. English speakers played again, under instructions to intentionally communicate color sample identity. Direct comparison of senders’ samples and receivers’ choices revealed categorical understanding of colors without considering color naming data. Although Somali-speaking senders provided fewer color terms, interpersonal Mutual Information (MI) calculated from color naming data was similarly below optimal for both groups, and English-speaking dyads’ MI did not improve with experience. Both groups revealed superior understanding of color terms because receivers showed better exactly-correct selection performance than was predicted by simulation from their senders’ color-naming data. This study highlights limitations on information-theoretic analyses of color naming data.

## Introduction

**Figure 1:**
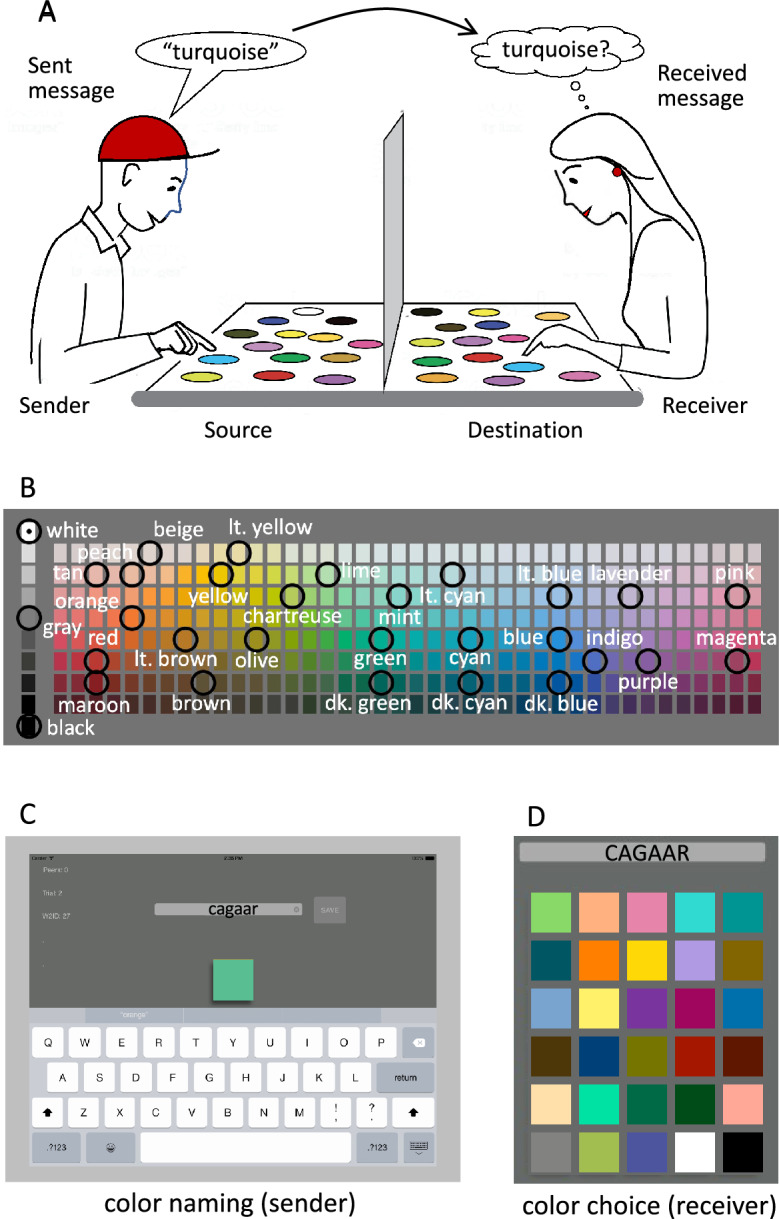
Methods and stimuli. (**A**) the Color Communication Game. (**B**) the colors used in this study, shown with the corresponding Munsell color samples from the World Color Survey. (**C**) The sender (a Somali speaker in this example) views a sample from his source set of samples and sends a message (either a single color term, or “I don’t know”) to the receiver. (**D**) The receiver hears or sees the term then selects, from her destination set of color samples, the one she thinks the sender intended to communicate. Neither player can see the other’s samples (shown by a baffle in A). The message improves the receiver’s likelihood of selecting the correct sample, but it does not guarantee success. See Sect. “Experimental methods” and Supplement for further details.

There is a large literature, spanning 140 years, on how color terms come to be associated with the material properties of objects. A robust finding from this research is that there is great diversity among world languages in how many color terms are in their lexicons, but that there are striking regularities across languages in the deployment of their color terms across a standard palette of test colors [reviewed by^1,2^]. Recent research suggests that these regularities might be related to a culturally universal need for efficient communication^3–5^, within the constraints imposed by the characteristics of human color vision^6,7^. However, we have shown that there is also considerable diversity in individual color vocabularies within languages^8,9^. This within-language diversity would seem likely to impede accurate communication among speakers. Furthermore, color naming experiments can only reveal how an informant chooses to communicate about a particular set of colors at a particular point in time, and they may miss the informant’s full understanding of their language’s color terms. We have previously developed an analytical tool based on information theory^10^, which we call the Color Communication Game (Fig. 1A), for making cross-cultural comparisons of efficiencies in color communication based on color naming. Here, we explore the “gap” between production and knowledge by having subjects actually play the Color Communication Game. To probe for possible cross-cultural differences in this gap, we compared informants speaking Somali and American English, two languages whose color lexicons are known to differ in size.

### The color communication game

Our Color Communication Game is patterned after the classic communication system proposed by Shannon & Weaver^11^ (see also^12,13^ for similar and^14^ for related approaches). Recent work by others has also been based on information-theoretic analyses of existing color-naming data sets [e.g.^3,15^]. Shannon & Weaver’s system consists of an information source (here, a set of color samples) and a sender, who encodes one of the samples in a color message (a color term) which is transmitted to a receiver. The receiver decodes the message and identifies the intended sample at its destination (Fig. 1A; we arbitrarily refer to the “players”—i.e., the sender and receiver—using masculine and feminine pronouns, respectively). In the present study, players participated in the communication game, which we implemented on calibrated iPads. Players first served as senders by naming each color in a test palette (Fig. 1B,C) and then, as receivers, they used sender messages (including their own) to guess the corresponding source colors (Fig. 1D). To closely follow the methods in the color naming literature, we did not tell the senders that they were to identify the intended samples as receivers until the second round of the game. To find out what effect knowledge of the game had on performance, we debriefed the English-speaking players after their first round as “naïve” players. Then the English speakers played the game a second time as “experienced” players (see Supplement, “instruction scripts”).

The color-naming data from the “sender” phase of the experiment were analyzed using the information-theoretic metric Mutual Information (“MI”, measured in bits). Subjects’ choice data from the “receiver” phase of the experiment were examined by simulation. Both quantitative approaches (see Sect. “Quantitative methods”) rely on two assumptions:

Assumption #1: Given a sender’s message, *m*, all colors that the receiver calls *m* are equally likely to be chosen by the receiver.

Assumption #2: Otherwise, in the absence of any understandable message, all palette colors are equally likely to be chosen by the receiver.

In the special case where the sender and receiver are the same person, we assumed that the message color terms correspond identically with the receiver’s vocabulary, so Assumption #2 will be irrelevant.

To summarize, we compared the predictions of communication efficiency based on information-theoretic analysis of color-naming data to choice performance when the Color Communication Game is actually played. Thus, we “closed the loop” by asking our Somali-speaking and English-speaking participants to actually play the Color Communication Game that underlies both the information-theoretic approach and the simulation approach to understanding communication about color. This allowed us to compare these analyses to the observed choice performance, which may depend on other factors not specifically captured by information theory.

## Results

### Color-naming data

Thirty-four Somali-speaking senders and 63 Somali-speaking receivers formed 63 dyads, with each receiver playing opposite one of the 34 senders. They used a total of 74 distinct color terms (average 10.5, ± S.D. = 2.2 terms/player). Thirty-one English-speakers played in groups of 2 to 4 individuals, forming 70 dyads, with each receiver playing opposite the other members of the group (see Sect. “Experimental methods” for further details). The English data sets contained 94 terms in the first (naïve) round of the game and 120 terms in the second (experienced) round, with individual vocabularies averaging 20.9 ± 4.5 and 25.6 ± 2.9 terms/subject, respectively. These total numbers of terms are large compared to studies where low-consensus terms are often considered lexical “noise,” and are excluded by considering only responses above some threshold frequency [e.g., about 80% in the WCS, ref.#1], but small compared to studies where multiple terms are allowed ad lib.^16^.

**Figure 2:**
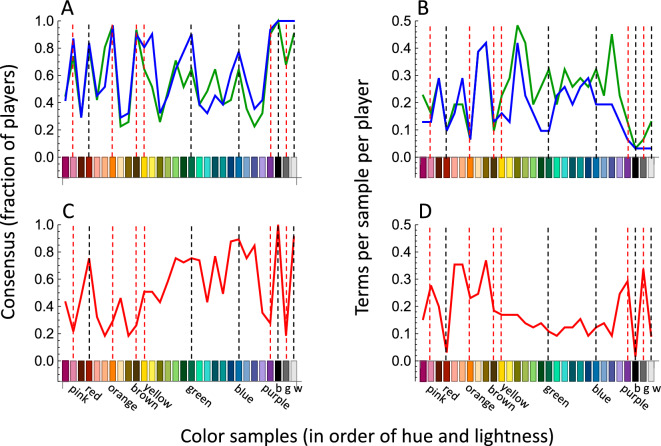
Comparison of color naming results for English (naïve: blue, experienced: green) and Somali (red) color terms, as functions of color sample, sorted in order of hue, then lightness. Color terms below abscissas in C and D correspond to English basic color terms. Black dashed lines highlight samples named with high consensus in both English and Somali. Red dashed lines indicate colors named with high consensus in English but not Somali. (**A**, **C**) consensus: the fraction of players who used the most common term for each sample; consensus varied by hue (F_1,86_ = 7.649 *p* = 0.007; hue accounted for 8.2% of the variance in consensus), but the average consensus did not differ across data sets (F_2,86_ = 0.888, NS; data sets accounted for 2% of the variance in average consensus). (**B**, **D**) number of distinct terms per sample, averaged across players; the number of terms per sample varied by hue (F_1,86_ = 4.049, *p* = 0.047; hue accounted for 4.2% of the variance in term number) and across data sets (F_2,86_ = 4.097, *p* = 0.020; data sets accounted for 13.9% of the variance in term number).

Graphs in Fig. 2A,C plot consensus—defined here as the fraction of English-speaking or Somali-speaking players who used the most common term for a sample in our test palette—as a function of test palette color. The fraction of terms used by fewer than four players was similar for English (naïve: 0.374; experienced: 0.341) and Somali (0.396). Graphs in Fig. 2B,D plot a complementary statistic: the mean number of terms that senders offered for each sample. English consensus among naïve players (blue lines) was high, and word count correspondingly low, for those samples chosen to be good examples of English basic color categories (dashed lines and abscissa labels). Consensus and word count were variable for the other, non-basic samples. Remarkably, the patterns of consensus from English-speaking participants as naïve and experienced senders (blue and green lines, respectively) were similar, even though experienced senders provided somewhat more terms throughout the palette region between yellow and violet. Evidently, the individual additional terms were used by few players, and did not significantly improve the overall level of consensus.

Somali consensus was also high (and word count low) for some basic color categories (red, green, blue, black and white: dashed black lines in Fig. 2D). For pink, orange, and purple and gray, the complementary pattern also held: consensus was low and word count was high. For brown and yellow, consensus was not high, and word count was in the middle of the Somali range, so no single term particularly dominated for those samples. As with English, Somali consensus for the remaining non-basic palette colors was variable, owing primarily to complementary variability in word count.

These findings obtained with our 30-color palette largely replicate those of our previous studies of English^17^ and Somali^18^, which were obtained using much larger test palettes. As is true of most world languages^1,19^, those studies showed that the English and Somali color lexicons were generally similar in semantic structure, although the Somali lexicon was smaller, and a minority of informants used a common term for green and blue (Figs. S2, S3). These findings are also compatible with Maffi’s linguistic comparison of English and Somali^20^.

### Communication with oneself

**Figure 3:**
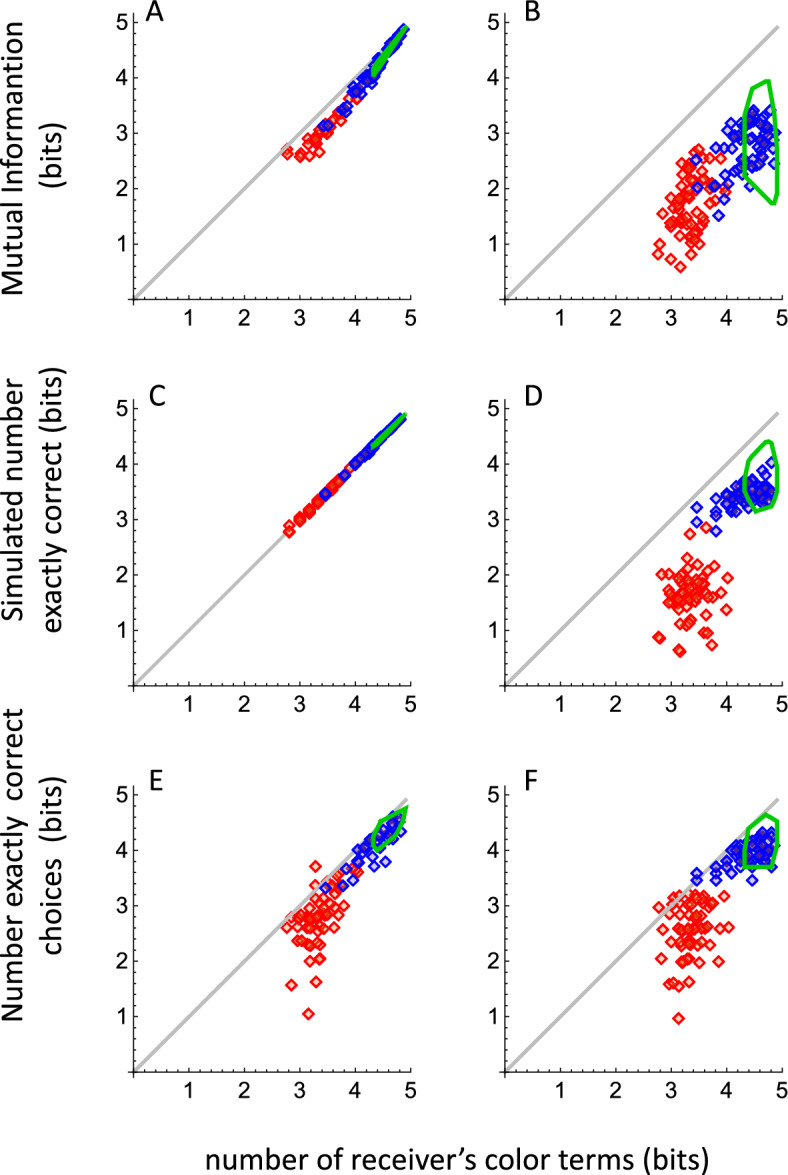
Mutual Information, simulations and results, as a function of the number of color terms in the receiver’s vocabulary. Somali-speaking players: red diamonds. English-speaking players: naïve, blue diamonds; experienced, green convex hulls. All data in log_2_ units (bits). Columns (**A**, **C**, **E**): self-communication; columns (**B**, **D**, **F**): dyadic communication. First row: Mutual information from color-naming data. Gray lines, theoretical maximum MI from Eq. (4). Second row: Simulated correct performance from color-naming data. Third row: Empirical exactly-correct choice performance. The major diagonal is not a theoretical maximum for exactly-correct performance, because a player could do better by chance.

The system shown in Fig. 1 is simpler if the sender and the receiver are the same person. For example, in everyday life, a person might write down a color term as a mnemonic aid for later recall of a colored object. The color terms provided by the player as sender often covered many color samples. By Assumption #1, any sample communicated by a given color term should be equally likely to be chosen by that same player as receiver. If we assume that all the terms contained in the player’s vocabulary during the sending phase, including “I Don’t Know” (DK), were still in the player’s vocabulary during the receiving phase, we do not need Assumption #2, because the rational player should treat DK like any other color term. That is, when she sees DK, she should choose randomly among the samples she had previously called DK, and she will not need a special strategy for choosing those DK samples. Figure 3A shows the calculated MI for self-communication. Throughout this report, individual data and predictions for the naïve experiments are shown by data points (Somali: blue; English: red). Experienced English data are summarized by green loops, which are the convex hulls around the individual data.

The major diagonal in Fig. 3A plots the highest possible value of MI for each number of terms in the receiver’s vocabulary. This will occur when the self-communicator’s color terms partition the test palette into equal-sized categories (Eq. (4); see also^21^). The calculated MI values from the Somali and English color-naming data fell on a common trend that was slightly below the constraint line because the sizes of the categories varied from color to color within players.

All English speakers but one used more terms in the second round than in the first (green loop in Fig. 3A; mean increase = 0.31 bits; bootstrapped 95% range: 0.24–0.35). All but one improved their level of MI (mean increase = 0.40 bits; 95% range: 0.29–0.53 bits), and the first and second self-communication MI data sets fell on the same general trend. Thus, using more terms in the second round helped players perform the self-communication task.

### Interpersonal communication

Mutual Information for interpersonal communication, within the Somali-speaking dyads and within both naïve and experienced English-speaking dyads, consistently fell about 1.7 bits below the constraint line, and all three data sets were more variable than the self-communication data (Fig. 3B). Variability increased because senders and receivers often used different terms, so a receiver frequently saw color terms that were not in her vocabulary. Even when the sender’s and receiver’s terms were identical, they may have been associated with slightly different stimulus sets. For example, one player might use “green” to name three greenish samples, whereas another player might use “green” for just one sample.

In contrast to the self-communication results, only 57% (95% range: 46–69%) of English-speaking dyads improved their level of MI in the second round of play. Comparing the naïve data (Fig. 3B, blue data points) to the experienced data (green loop), the mean increase was 0.13 bits (bootstrapped 95% range: –0.01 to  + 0.27, NS). This suggests that the additional terms in the experienced data set were not used with high consensus (Fig. 2A), and therefore were not usually in the receiver’s vocabulary and did not improve dyad MI performance.

## The color communication game

Color communication is not a naming game: the communication process is complete only after the receiver chooses which color sample she thinks the sender was viewing when he sent the color term message (Fig. 1A). We scored the results as “exactly” correct if the receiver chose the sample the sender intended when he sent the message. We scored the result as “categorically” correct if the receiver chose a sample from her own color category that matched the sender’s message term.

**Figure 4:**
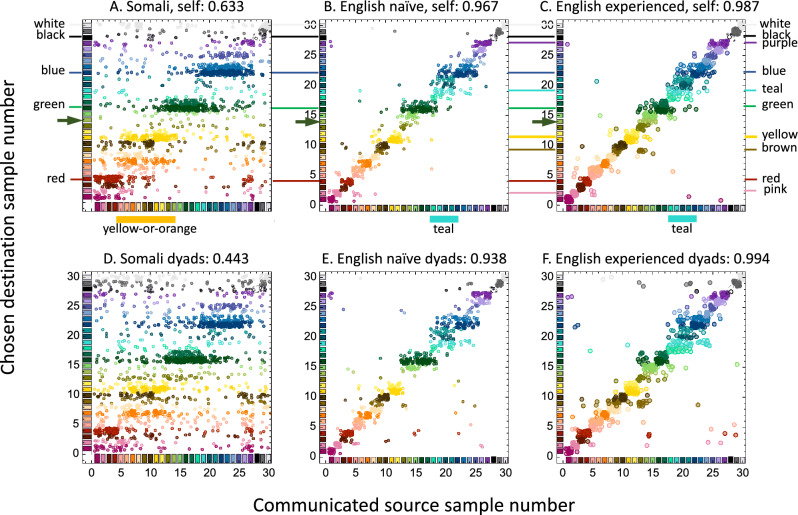
Accuracy of sample selection. Data points are the receiver’s chosen samples as a function of the sender’s intended samples. Data are color coded by the receiver’s color selections; positions jittered by less than ± 0.5 in two dimensions. Accurate selections, where the receiver chose the sample intended by the sender, fall on the major diagonal. (**A**–**C**), selections for self-communication, where the sender terms were provided by the receivers themselves; (**D**–**F**), selections for dyad communication, where the terms were provided by a sender other than the receiver. Numbers in the headings are Spearman’s rho, and all are highly statistically significant (*p* values near zero). Fiducial lines between panels (**A**–**C**) join clumps of samples associated the color terms in Fig. 2; arrow shows the chartreuse sample #14 (see Sect. “Discussion”); bars below panels (**A**–**C**) indicate groups of samples named by senders that receivers tended to respond to similarly.

Figure 4 shows the accuracy with which receivers chose the samples intended by their senders^14^. Each dot was from a single trial, where the choice of the receiver (y-axis value) was associated with the sample named by the sender (x-axis value). Exactly-correct performance will fall on the major diagonal. The sender and receiver data in every panel were highly correlated, which indicates that players were engaged in the task. In general, the Somali data showed more dispersion than the English data did, and the Somali dyad data showed more dispersion than the Somali self-communication data.

A remarkable feature of these diagrams is that the data often appeared in horizontal clumps: a relatively wide domain of multiple samples named by the sender was commonly associated with a relatively narrow range of samples chosen by the receiver. Some samples were chosen frequently (e.g., the dark green sample, #16), whereas other samples were chosen rarely (e.g., the chartreuse sample, #14), even though each sample in the stimulus set was named equally often. The horizontal lines in Fig. 4 are added for reference. They show that many of the clumps were located in regions of high consensus in Fig. 2, but others, notably the teal clump in English, were not convincingly present in Fig. 2. Furthermore, some of the clumps line up vertically into groups. For example, the clumps of yellow and orange data points were grouped in the Somali data (orange bar below panel A), because they corresponded to the same domain of sender samples. There was a similar group of teal and light blue clumps in the English data (cyan bar below panel B). The experienced English data show less evidence of clumping because the domain for each color term was only a few samples wide, but the choices for teal nevertheless align into their own group (cyan bar below Fig. 4C). The clumps and groups of clumps in these choice data are reminiscent of many of the lexical color categories found previously using only color naming data^17,18^. However, no lexical analysis was used here: these diagrams show the receivers’ understanding of the color terms communicated by the senders, as expressed by the receivers’ choices, regardless of what color terms the senders might use. Indeed, the group of clumps corresponding to teal (in English) were named using 19 different color terms, yet the receivers chose the samples appropriately, and therefore understood the senders very well. These clumps and groups of clumps were wholly serendipitous, and bear following up with stimulus sets designed to reveal them even more clearly.

### Exactly-correct choice performance

Figure 3C,D show the choice results predicted from simulations based on Assumptions #1 and #2, above (see Sect. “Quantitative methods”). The best predicted performance was the simulated number of correct responses for self-communication (Fig. 3C), which fell on the major diagonal. This prediction follows from the design of the experiment, where the player named each sample exactly once. If a term occurred *m* times in the self-communicating player’s lexicon, it was chosen *m* times, each time with a probability of 1/*m*, and the player scored a “1” for that term. Thus, random behavior by the self-communicating player in the face of uncertainty did not reduce the simulated level of performance below the major diagonal. When exactly-correct performance for communication within dyads was simulated from the naïve players’ vocabularies, predicted performance was 0.92 bits (English) or 1.73 bits (Somali) lower than for self-communication (Fig. 3D).

The empirical number of exactly-correct choices for self and dyadic communication generally fell below the major diagonal (Fig. 3E,F). All but two English-speaking players showed higher self-communication scores in the second round (Fig. 3E), for a mean increase of 0.39 bits (bootstrapped 95% range: 0.29–0.49 bits). Eighty-two percent of dyads (95% range: 74–92%) improved their exactly-correct performance in the second round (Fig. 3F; average improvement = 0.22 bits, 95% range: + 0.16 to  + 0.28). Thus, dyads showed a somewhat greater improvement for exactly-correct performance than for MI. Unlike MI, exactly-correct performance reflects both the increase in the number of terms used and the receiver’s understanding of more terms than they offered in the sender phase.

**Figure 5:**
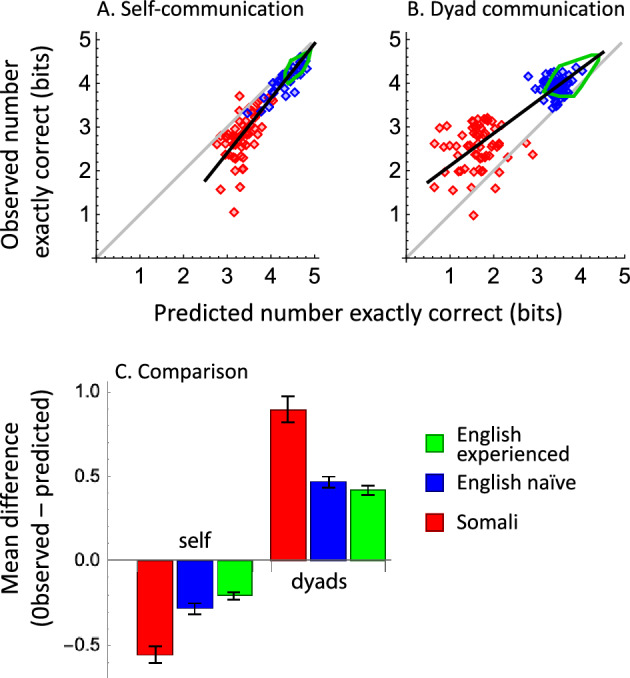
Exactly-correct identification performance as a function of predicted performance based on simulations. (**A**) self-communication performance is not as good as the predicted level of performance for self-communication based on simulations; because the self-communication prediction falls exactly on the major diagonal (Fig. 3C), the data in panels 5A and 3E are identical. (**B**) dyad communication performance is better than predicted. Lines in A, B are fitted descriptively to the medians of the data, and have no theoretical significance. (**C**) mean gaps, ± SEM, between the predicted and observed results in (**A**, **B**).

Figure 5 compares the observed performance from Fig. 3E,F directly to the predicted performance from the simulations in Fig. 3C,D. The number correct for self-communication is predicted to fall on the major diagonal, but the individual results in Fig. 5A and the left group of bars in Fig. 5C show that both groups of self-communicators performed below the predictions. This could happen even though a receiver knew her own terms, which she previously provided as sender, if the exact range over which each term applied varied over time. For example, an English speaker certainly knows the term “green” equally well as sender and receiver, but when she is the receiver, she might have forgotten exactly which samples she had previously called “green”. This result is different from the MI results (Fig. 3A), because the MI calculation involved only the behavior of the player at the instant the color sample was named, so no memory was involved.

Communication performance within dyads was better than the simulations predicted for both groups (Fig. 5B,C-right bars). This occurred because receivers understood more synonymous terms than the single terms they were allowed to provide as senders (c.f.^22^). This was especially true of Somali dyads, whose performance was 1.9 times better than predicted. Unlike human communicators, the simulations treated different but synonymous terms as “unknown to the receiver” in accordance with Assumption #2, so the simulations underpredicted human performance.

### Choice strategies

The two assumptions that underlie both the information-theoretic analysis and the simulations can be evaluated directly by examining players’ choice behavior.

#### Assumption #1

**Figure 6:**
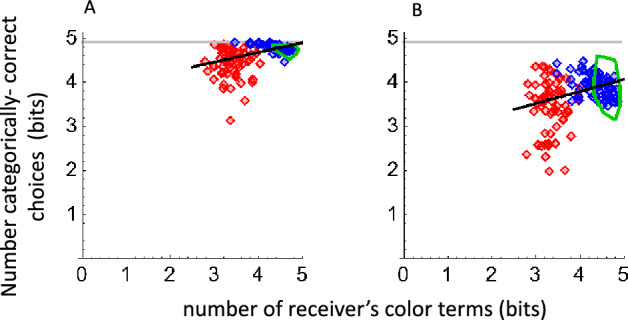
Categorically correct choices. Perfect performance is at log_2_^30^ for all sizes of the receiver’s vocabulary. The black lines are fitted descriptively to the three data set; they have no theoretical significance. (**A**) self-communication; mean values were 0.38 bits (Somali), 0.13 bits (English naïve) and 0.13 bits (English experienced) below perfect. (**B**) communication within dyads; mean values were 1.45 bits (Somali), 0.96 bits (English naïve) and 1.03 bits (English experienced) below perfect.

At a minimum, a receiver who obeys Assumption #1 should always choose a categorically-correct sample if the message term is in her vocabulary. For example, if the message term was “green,” and if “green” was in the receiver’s vocabulary, she should choose a sample that she had previously called “green”. A self-communicating receiver with perfect memory for her color terms from the naming phase, and who remembered the exact distribution of her color terms across the samples, should perform perfectly by this standard. In fact, the categorically-correct scores for self-communication (Fig. 6A) did not reach perfect performance. This result, like the self-communication results in Fig. 5, suggests that the domains of the color terms probably shift over time. The categorically-correct scores for communication within dyads (Fig. 6B) were lower, presumably because the receivers did not always recognize their senders’ terms, and the categories defined by the sender’s and receiver’s corresponding terms might differ.

Assumption #1 more rigorously predicts that receivers should distribute their categorically-correct choices in an unbiased way within the categories defined by the color terms they recognize. That is, the range of choices for a given color term should be approximately the same as the domain of the samples called by that term. This was clearly not true of the Somali and the naïve English data (see also Fig. S4). Much of the naïve data (Fig. 4A,B,D,E) appeared in horizontally-oriented clumps, which were bounded above and below by regions of sparseness. By comparison, the clumps in Fig. 4C,F were approximately square, showing that experienced players chose their samples relatively uniformly. This might have occurred because the number of terms (25 for each experienced player) approached the number of samples, so the categories were very small, leaving little room for free choice of samples. Or, it might have occurred because the experienced receivers chose to obey Assumption #1. Further experiments, involving many more samples, would be required to resolve this issue.

#### Assumption #2

**Figure 7:**
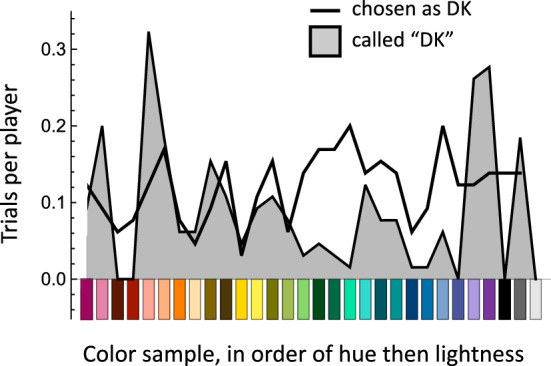
Samples named “I don’t know” and samples chosen on trials where DK was the message color term in the Somali data set. The distribution of DK messages varied significantly across the sample set (F_29,1410_ = 4.747, *p* < 0.001; the color sample factor accounted for 8.9% of the variance in the samples called DK), but the choices of samples in response to DK were approximately constant (F_29, 1410_ = 0.995, NS; color sample accountedfor 2% of the variance in DK choices). Furthermore, participants’ choices of DK samples were unrelated to their personal use of DK (partial correlation: r = 0.001, *p* = 0.97, NS).

Players of the color communication game were allowed to respond “I don’t know” (DK) if they did not know an appropriate color term, thereby providing no understandable message. Somali-speaking players used DK on 11.8% of the color-naming trials (shaded region of Fig. 7). These data allowed us to evaluate Assumption #2 for the Somali data set. The senders’ use of DK was unevenly distributed across the color palette (DK was never used for the maroon, red, dark blue, white, or black samples). However, consistent with Assumption #2, the selections made by the receivers in response to DK were approximately uniformly distributed across the color palette, and receivers’ choices were not associated with their personal use of DK. English speakers never used DK, so we cannot examine Assumption #2 for them.

## Discussion

This study of the Color Communication Game was motivated by two related issues. First, our previous analyses of the WCS revealed considerable within-language diversity in color naming^8^. This seemed at odds with prior information-theoretic studies, which concluded that the color terms in a language are shaped by the need for efficient communication^4,23^. Second, most previous research on color terms has been concerned with color-naming data, but it seemed likely that, while color-naming provides information about the inventory of color terms in a language community, it cannot reveal the entirety of an individual’s lexical knowledge about color.

To address these issues, we asked English-speaking and Somali-speaking participants to play the Color Communication Game using a test palette of 30 colors that was designed to be large enough to elicit the major patterns of color naming that are known to exist in Somali^18^, English^17,24^ and other world languages^1,25^, yet small enough to measure subjects’ lexical knowledge in a brief color identification experiment. We calculated MI from the color-naming data only, then we compared the exactly-correct and categorically-correct choice performance of the receivers to the results of computational simulations. When plotted against the number of terms in the receivers’ vocabularlies, the empirical results in Fig. 3A,B,E,F and Fig. 6 were lawfully arrayed, with larger vocabularies being associated with higher MI scores and better choice performance.

The values of MI for English-speaking and Somali-speaking dyads were consistently below the constraint line (the major diagonal), generally replicating the results from our previous work (Table S1). We attribute the cross-cultural differences in MI we observed here and in the WCS^10^ to variation across languages in the number of lexically salient regions in the WCS chart. Lindsey & Brown^17^ identified 20 such regions for American English[c.f.,^22,26^], while Brown et al.^18^ identified only 11 regions for Somali. The large number of DK responses in the present Somali data set reinforces Levinson’s claim that a language’s lexical color categories do not always carve up color space exhaustively^27^. Likely because we allowed DK, the fraction of terms used by fewer than four players was similar for English and Somali.

Exactly-correct performance within dyads was higher than was predicted from our simulation analysis based on the color term messages (Fig. 5). This gap between prediction and performance suggests that the mapping between terms and samples is many-to-one; that is, people know more color terms for a given sample than they reveal in a standard color-naming study, where only single terms are allowed. It also highlights the point that traditional color naming studies are an imperfect measure of participants’ knowledge about colors. Interestingly, self-communicators’ choice performance was lower than the simulations predicted, which is in the opposite direction from the result for communication within dyads. This opposite gap reveals an important feature of color cognition. We can assume that players generally recognize their own color terms when they are receivers, but it seems likely that the mapping between terms and samples might shift between when the player served as sender and when she made her choices as receiver. Casasanto & Lupyan^28^ have emphasized the fluidity of categories across different contexts, while Yee and Thompson-Schill^29^ highlight temporal factors in this process. An additional factor may be limitations on human memory for colors or for their associated color terms^30^. Any of these time-varying factors could also explain why players did not perform perfectly when their performance was scored as “categorically correct” (Fig. 6A).

These two gaps illustrate the importance of two roles that color terms may serve: self-communication, where they may serve as mnemonic aids, and communication about color with other members of the language community^14^. Very precise terms, each of which names few colors, may be useful to an individual (within the limits of their own memory capacity), but they may not be needed when communicating with others, where a categorically-correct understanding of the speakers intention will often suffice. We speculate that the color terms that were originally intended for self-communication may come to be used more often for communication with others, and they may eventually become the language’s basic color terms.

There is a large literature on language games, which often aims to understand how lexicons come into existence. Typically, these games are played for multiple rounds, and players develop lexicons and pragmatic strategies to communicate about novel stimuli[e.g.,^31,32^]. The present project was specifically aimed at color terms as they are used and understood today, in everyday life. Naïve Somali-speaking and English-speaking players named the colors spontaneously, then they tried to determine what colors the message terms denoted. We repeated our English game a second time, with full instructions, to examine the behavior of players who intentionally communicate the identity of a sample to a receiver. The self-communication results in the naïve and experienced data were closely associated, suggesting that whatever combination of strategy and practice affected their play in the second round improved the performance of the group by increasing the number of terms in the players’ vocabularies. In contrast, the dyad results showed much less improvement. This suggests that the increased number of terms in the second round did not define additional color categories, and therefore did not allow participants to communicate more effectively.

Figure 4 compares the samples intended by the sender directly to the choices made by the receiver, without involving any consideration of the numbers or identities of the color terms. In most cases, the chosen samples were high in chroma, which might suggest that perceptual salience may have dominated receivers’ choices^33^. However, this seems unlikely, because the chartreuse sample (green arrow in Fig. 4) was rarely chosen, even though its chroma was among the highest in our palette. These selections were also reminiscent of the red, green, blue and yellow unique hues, which are similar for English and Somali^34^, and which, for English at least, are close to the corresponding focal colors. However, this similarity should not suggest a link to Hering’s theory of color appearance, which does not work well as an account of Somali color naming [^34^ see also]^13^.

More generally, the clumps and groups of clumps in Fig. 4 cluster around good examples of their respective color categories (see also Fig. S4), suggesting that subjects were biased to select category prototypes, in violation of Assumption #1. These strong preferences for specific color samples over others in the same lexical category suggest that people chose the (unmarked) prototypical stimulus when a category’s usual term appeared. For example, if the message was “blue,” a receiver might choose the prototypical blue sample rather than the dark blue sample, because if the sender had intended the (marked) dark blue sample, she may assume that he would have used “navy” as his message. Thus, players’ choice preferences are reminiscent of the listener-salience results of Frank & Goodman^35^.

We did not anticipate this pragmatic bias in receiver choice performance. Even though it shows the limits of Assumption #1, this result does not necessarily disqualify the information-theoretic approach to studying color communication, especially when only color-naming data are available. However, it does suggest that other approaches, perhaps combining information theory with Bayesian inference based on explicitly-identified priors to handle ambiguity in color communication [e.g.,^15,35^], may prove useful in future studies of the semantics of color.

## Experimental methods

This research was approved in advance by the Ohio State University Institutional Review Board for the protection of human subjects, and it conformed to the principles of the Declaration of Helsinki. Participants provided informed consent in their native language before the study began.

### Participants

A total of 97 monolingual speakers of the Somali language participated in this study (49 male, 48 female; average age, 30.8 years, range 18–60 years). They were recent arrivals into the U.S. and were recruited and tested in the waiting room of an Ohio State University medical facility where they received their medical screening before being further processed for immigration into the U.S. They were screened for color vision deficiency using the HRR plates, and none were found to be color deficient. All informed consent, instructions, and participation were handled verbally through experienced, native-speaking professional Somali language interpreters who were familiar with the experimental procedures. The 31 monolingual speakers of American English (5 male, 26 female) were color-normal faculty, students, and staff of the Ohio State University College of Optometry.

### Stimuli

Color samples (Fig. 1C,D) were presented on calibrated iPads via custom software, which controlled all aspects of the experiment. The 30 samples in the test palette (Fig. 1B) were chosen to elicit patterns of color naming that would approximately recapitulate those from prior studies of English^17,24^, Somali^18^, and other world languages[reviewed in 2]. The 30-color palette included black, white and gray, plus 27 chromatic colors that corresponded to samples in the WCS color chart (Fig. 1B). The chromatic colors were chosen to include 17 good examples of the known color categories in English and Somali, plus 10 additional colors. Simulations showed that these samples produced MI values that were similar to, and highly correlated with, the values obtained with the full WCS palette (see Supplement for further details). Table 1 shows swatches of the 30 color samples along with the most common terms provided by Somali-speaking and English-speaking participants.

**Table 1:**
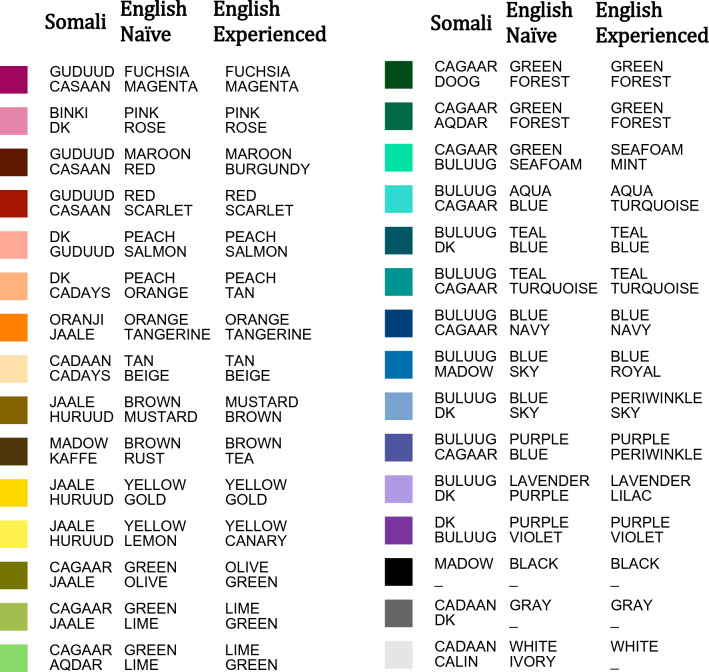
Swatches of colors and most common and second-most-common Somali and English color terms.

### Procedures

The 31 English-speaking participants were tested in groups of 2—4 players (mean = 3.1) using individual iPads linked in a local area network. The group sat at a large table with baffles provided to hide each participant's iPads from the other players in the group. Each individual was a sender and receiver for all members of their group, and the 31 English speakers formed 70 dyads. The terms they provided as senders were queued on their own iPads and transmitted asynchronously to the iPads of the other members (mean = 2.1) of their group, to be used as messages in the second phase of the game. English speakers participated in two rounds of the game. In the first round, they were naïve as to the purposes of the study; in the second round they were experienced and fully briefed.

In the first phase of the first round of the game, English-speaking participants were senders. They were instructed to type onto the iPad a single term for each sample from the palette of 30 test colors (source colors, Fig. 1B), which were presented one at a time in random order, without replacement (Fig. 1C). In the second phase of the game, the players, now as receivers, viewed each of their own messages, intermixed in random order with all the messages provided by the other players in their group. Thus, the number of trials in the second phase was 30 times the number of players in the group. Players were instructed to select, from a randomized array of the destination colors (Fig. 1D), the sample they thought the sender had been viewing when the color term was originally provided (see Supplement for instructions). The second round of the game was identical to the first, except that senders were instructed that the terms they provided would be used later to identify the color they were viewing in the first phase when they provided the name (see Supplement for instructions to subjects).

In a retrospective analysis, we examined the exactly-correct choice performance of the English speakers by group and performed a one-way Analysis of Variance on the results. For the self-communication data, F(9,21) = 1.02, *p* = 0.450; for the dyad data, F(9,60) = 0.842, *p* = 0.581. So neither the self-communication nor the dyad exactly-correct data varied significantly across groups.

Test procedures for the Somali players were the same as for the naïve English players, except for three changes. First, the assignment of participants to the sender and receiver roles was different. The first 34 Somali-speaking players provided only sender data, and their color terms were stored in iPad memory. In separate sessions, each of the last 63 players served as both sender of their own messages and receiver of the messages from one “other” sender, who was chosen randomly (with replacement) from the first 34 players. Thus, each of the senders participated in multiple dyads, each of the receivers participated in exactly one dyad, the 97 Somali-speaking players collectively formed 63 dyads, and there were 60 trials in the identification phase of the game. Second, the Somali players were tested through interpreters. In the sender phase, the 34 senders reported each color term verbally to the experimenter or interpreter, who typed the term into the iPad. In the receiver phase, each color term was read aloud to the participant by the interpreter. Third, Somali speakers were tested in a single round, as naïve players only.

## Quantitative methods

### Mutual information

Color-naming data were analyzed using the information-theoretic metric *Mutual Information*. Mutual information quantifies how much the sender’s message reduces the receiver’s uncertainty (*entropy*) about the identity of the sample that the sender intended to communicate. The calculation of entropy depends on two assumptions about the receiver’s behavior in the face of uncertainty, Assumptions #1 and #2, as described in the main test.

The entropy in the color communication game is determined by the number of color samples, *n*, in the test palette. By Assumption #2, in the absence of the sender’s messages, the receiver’s chance of guessing the sender’s color selection on any given trial is 1/*n*. The corresponding entropy, expressed in bits, is:

1 $$H\left( C \right) = - \mathop \sum \limits_{c \in C} p\left( c \right)log_{2} \left( {p\left( c \right)} \right)$$

If the receiver does not have any idea of the meaning of the sender’s message, then assumption #2 applies and the entropy is just *H*(*C*) = log_2_ (1/*n*). Mutual information, *I*, the reduction in entropy, given the sender’s messages is:

2 $$I\left( {C,M} \right) = H\left( C \right) - H\left( {C|M} \right)$$

In our analysis of color naming, we calculated mutual information by comparing the sender’s and the receiver’s color vocabularies. Our analysis was based on the following equation for mutual information:

3 $$I\left( {C_{R} ;C_{S} } \right) = \mathop \sum \limits_{s,r} p\left( {s,r} \right)log_{2} \left( {\frac{{p\left( {s,r} \right)}}{p\left( s \right)p\left( r \right)}} \right),$$

where Cr and Cs are the sender and receiver color selections, and are random variables. *p(s)* is the marginal probability that the sender will select sample *s*, and is always *1/n*, since by assumption all samples are equally likely to be selected (with replacement) by the sender. *p(r)* is the marginal probability that sample *r* will be selected by the receiver across all samples selected by the sender. *p(s,r)* is the joint probability that sample *r* will be selected by the receiver, given a) sample *s,* selected by the sender, b) the sender’s term for sample *s* (the “message”) and c) the receiver’s interpretation of that color term, based on her color vocabulary. By Assumption #1, if the sender’s color term is in the receiver’s vocabulary, then *p(s,r)* will depend on the number of times that term was used by the receiver to name colors in the test palette. For example, suppose the sender says “red” for the *i*^th^ sample in the test palette, and the receiver named k samples as “red”. In the receiver’s destination sample set, the receiver’s non- “red” samples are never selected (in which case, *p(s*_*i*_*,p)* = 0) and the receiver chooses randomly (by Assumption #1), one of her “red” samples (in which case p*(s*_*i*_*,p)* = 1/n*1/k.) In the case where the sender’s term is not in the receiver’s vocabulary (the receiver does not understand “red”), all *p(s*_*i*_*,r)* = 1/n*1/n = *1/n*^*2*^. If the sender’s color term is not in the receiver’s vocabulary, the receiver is assumed to not understand the sender’s message and, by Assumption #2, *p(s,r)* will reflect that all color samples are equally likely to be selected by the receiver.

We also used equation Eq. (3) to derive an expression for the theoretical maximum MI calculated in the Color Communication Game, given a color vocabulary containing w color terms. Receiver uncertainty reduction is greatest when (1) the sender and receiver vocabularies are identical, and (2) contain terms that are distributed equally across the *n* samples in the test palette. For this case, there are *n*^2^/*w* non-zero elements in the joint probability matrix *p(s,r)*, with each element equal to 1/n*w/n, and the marginal probabilities *p(s)* and *p(r)* are each *1/n*. Thus,

4 $$I_{max} = \frac{{n^{2} }}{w}\frac{w}{{n^{2} }}log_{2} \left( {\frac{{w/n^{2} }}{{1/n^{2} }}} \right) = log_{2} \left( w \right)$$

### Simulations of exactly-correct choices

For each of the source colors, the sender’s term was compared to all the receiver’s color terms. If the sender’s term was in the receiver’s set of color terms, the simulator uniformly-randomly selected one of the destination colors associated with that term. If no receiver term matched the sender’s message, the simulator uniformly-randomly selected a destination color from among all 30 palette colors. A hit was scored if the chosen destination color matched the source color. Simulated exactly-correct choices equaled the number of hits summed across all source colors, averaged over 100 runs of the simulation.

Whereas the calculation of MI is based on the assumption that the sender chooses each sample independently (i.e., with replacement), the senders in this experiment chose each sample exactly once (i.e., without replacement). When the simulations described above for Fig. 3 were repeated with replacement, there was no significant change in the results (Somali: t = 0.451, *p* = 0.653; English: t = 0.415, *p* = 0.680).

### Supplementary Information

Supplementary Information.

## Data Availability

The datasets generated and/or analyzed during the current study are available in the Dryad repository: https://datadryad.org/stash/share/Xs0GiMMmM_FYHKCp6_Q31y_VAGS9WmN7rqn316jTiAk.

## References

[1] Kay P (2010). The World Color Survey.

[2] Lindsey DT, Brown AM (2021). Lexical color categories. Annu. Rev. Vis. Sci..

[3] Zaslavsky N (2018). Efficient compression in color naming and its evolution. Proc. Natl. Acad. Sci. U.S.A..

[4] Gibson E (2019). How efficiency shapes human language. Trends Cogn. Sci..

[5] Kemp C, Xu Y, Regier T (2018). Semantic typology and efficient communication. Annu. Rev. Linguist..

[6] Jameson KA, D'Andrade R, Hardin CL, Maffi L (1997). It's not really red, green, yellow, blue: an inquiry into perceptual color space. Color Categories in Thought and Language.

[7] Zaslavsky N (2019). Color naming reflects both perceptual structure and communicative need. Top. Cogn. Sci..

[8] Lindsey DT, Brown AM (2009). World color survey color naming reveals universal motifs and their within-language diversity. Proc. Natl. Acad. Sci. U.S.A..

[9] Webster MA, Kay P, MacLaury RE, Paramei GV, Dedrick D (2007). Individual and population differences in focal colors. The Anthropology of Color.

[10] Lindsey DT (2015). Hunter-gatherer color naming provides new insight into the evolution of color terms. Curr. Biol..

[11] Shannon CE, Weaver B (1949). The Mathematical Theory of Communication.

[12] Baddeley R, Attewell D (2009). The relationship between language and the environment: information theory shows why we have only three lightness terms. Psychol. Sci..

[13] Gibson E (2017). Color naming across languages reflects color use. Proc. Natl. Acad. Sci. U.S.A..

[14] Lantz D, Stefflre V (1964). Language and cognition revisited. J. Abnorm. Psychol..

[15] Twomey CR (2021). What we talk about when we talk about colors. Proc. Natl. Acad. Sci. U.S.A..

[16] Lin, H. *et al.* A cross‐cultural colour‐naming study. Part I: Using an unconstrained method*.**Color Res. Appl.* **26**(1), 40–60 (2001).

[17] Lindsey DT, Brown AM (2014). The color lexicon of American english. J. Vis..

[18] Brown AM, Isse A, Lindsey DT (2016). The color lexicon of the Somali language. J. Vis..

[19] Berlin B, Kay P (1969). Basic Color Terms: Their Universality and Evolution.

[20] Maffi L (1990). Somali color term evolution: grammatical and semantic evidence. Anthropol. Linguist..

[21] Cover TM, Thomas JA (2012). Elements of information theory.

[22] Derefeldt, G. & Swartling, T. Colour concept retrieval by free colour naming. Identification of up to 30 colours without training. *Displays* **16**(2), 69–77 (1995).

[23] Regier, T., Kemp, C. & Kay, P. Word meanings across languages support efficient communication. In *The Handbook of Language Emergence*, (eds MacWhinney, B. & Grady, W.O.) 237 (Wiley, 2015).

[24] Mylonas D, MacDonald L (2016). Augmenting basic colour terms in English. Color. Res. Appl..

[25] Lindsey DT, Brown AM (2006). Universality of color names. Proc. Natl. Acad. Sci. U.S.A..

[26] Griffin LD, Mylonas D (2019). Categorical colour geometry. PLoS ONE.

[27] Levinson SC (2000). Yélî Dnye and the theory of basic color terms. J. Linguist. Anthropol..

[28] Lupyan G, Casasanto D (2015). Meaningless words promote meaningful categorization. Lang. Cogn..

[29] Yee E, Thompson-Schill SL (2016). Putting concepts into context. Psychon. Bull. Rev..

[30] Olkkonen M, Allred SR (2014). Short-term memory affects color perception in context. PLoS ONE.

[31] Hawkins RD, Frank MC, Goodman ND (2020). Characterizing the dynamics of learning in repeated reference games. Cogn. Sci..

[32] Müller TF (2021). Colour terms: native language semantic structure and artificial language structure formation in a large-scale online smartphone application. J. Cogn. Psychol..

[33] Lucy JA, Schweder RA (1979). Whorf and his critics: Linguistic and nonlinguistic influences on color memory. Am. Anthropol..

[34] Lindsey DT, Brown AM, Lange R (2020). Testing the cross-cultural generality of hering’s theory of color appearance. Cogn. Sci..

[35] Frank MC, Goodman ND (2012). Predicting pragmatic reasoning in language games. Science.

