# Supplementary material for: The color communication game
Source: Sci Rep. 2023 Sep 25;13:16006. doi: 10.1038/s41598-023-42834-3 (PMC10520057; doi:10.1038/s41598-023-42834-3)
Supplement: Supplementary file 1 — Supplementary Information. [file 41598_2023_42834_MOESM1_ESM.docx]

SUPPLEMENTARY INFORMATION

1. **Color Palette**

Much of the previous work in the field of color naming is based on the 330 colors in World Color Survey (WCS) palette. These Munsell sample sets include 320 chromatic colors plus 10 achromatic colors (colored and neutral swatches in Figs. 1B and S1).

The palette of colors used in the present study was based on our own previous work on color naming in Somali [1] and (American) English [2]. In those studies, participants provided a single, monolexemic color term for each sample from a large stimulus set. The English study used a palette of all 330 WCS colors, and the Somali study used a 140-color “checkerboard” subset of the chromatic WCS colors plus 5 achromatic colors. The 30-color test palette used here consisted of black (approximately Munsell N0), white (N10) and gray (N4.5), plus 27 chromatic samples (black circles in Fig. 2 of the main text and shown in Fig. S1). In this supplement, we show that those colors are well chosen to include good examples of the major color categories that exist in Somali and English.


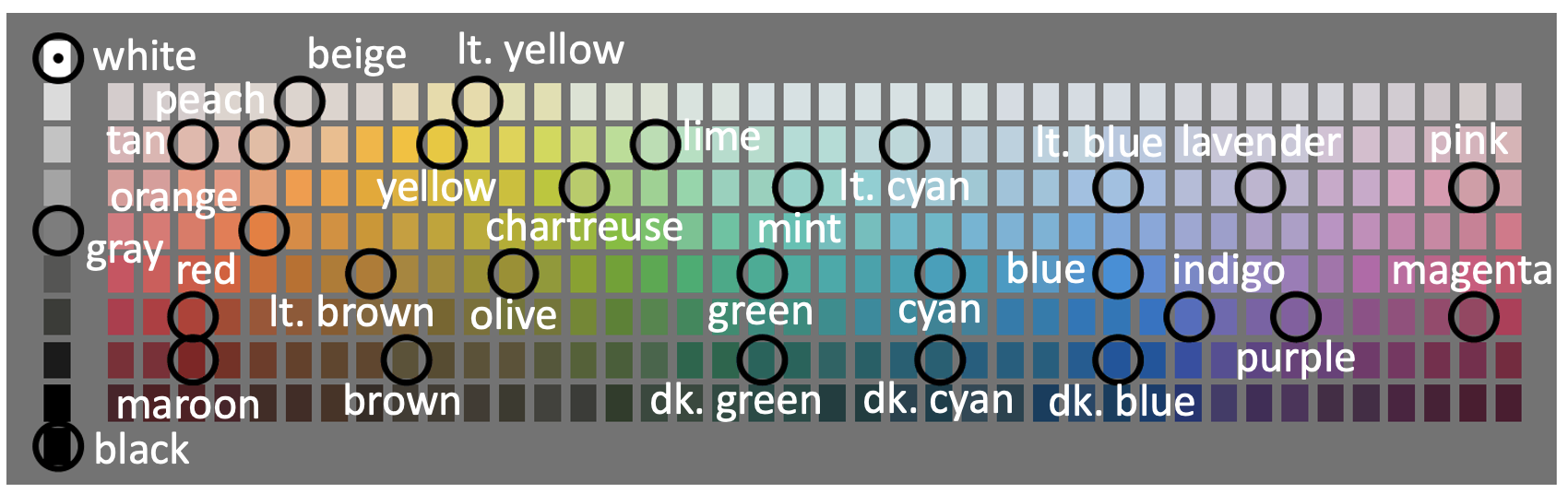


Figure S1. Locations of 30 test palette colors in WCS color chart, along with names used to refer to the stimuli in the main text.

Figures S2 and S3 compare the 27 chromatic colors used here to the lexical color categories from our previous work on English and Somali. In those previous studies, we defined a lexical color group (LCG) to be a set of WCS tiles that were given the same name by an informant. Then we used cluster analysis to assemble these LCGs into categories based on the sizes and locations of the LCGs within the color chart, irrespective of the actual color term given to an LCG by a particular informant. Cluster analysis of English-speaking informants’ lexical color groups (LCGs) revealed 17 categories (Fig S2).

One advantage of this cluster analysis approach was that lexical color categories could be identified even when the LCGs in those categories received different labels from different informants [3]. The English category TEAL, for example, was called by many different names: teal, turquoise, aqua, jade, etc.

Each panel in Fig. S2 shows the normalized sum of the LCGs in each lexical color category in English. The intensity of each small rectangle in the diagram represents the relative number of times the corresponding tile occurred in the LCGs assigned to that category by cluster analysis. Eight of these categories are easily identified with eight of the basic chromatic color categories of Berlin & Kay [4]: RED, YELLOW, GREEN, BLUE, BROWN, ORANGE, PINK, and PURPLE, coded as the colors of the tiles in Fig. S2. Informants usually named the tiles in the LCGs in these categories with the corresponding high-consensus Basic Color Terms of English: red, yellow…, etc.

Our cluster analysis of English also revealed 9 additional lexical color categories. These were not named with the same degree of consensus as the basic categories, but informants’ LCGs nonetheless frequently fell into one of these privileged regions of the WCS chart. These are identified in Fig. S2 as PEACH, TEAL, LAVENDER, MAROON, GOLD, BEIGE, MAGENTA, LIME and OLIVE.

On the basis of these results, 17 of the 27 chromatic colors in our test palette were chosen as representatives of the corresponding high-salience regions of the WCS color chart. The remaining 10 colors were chosen from regions of the WCS chart that we thought likely to be less lexically salient. Thus, by this choice of 30 test colors, we expected to evoke patterns of color naming from our English participants that replicated the basic findings of our original study of English color naming (Fig. S2).


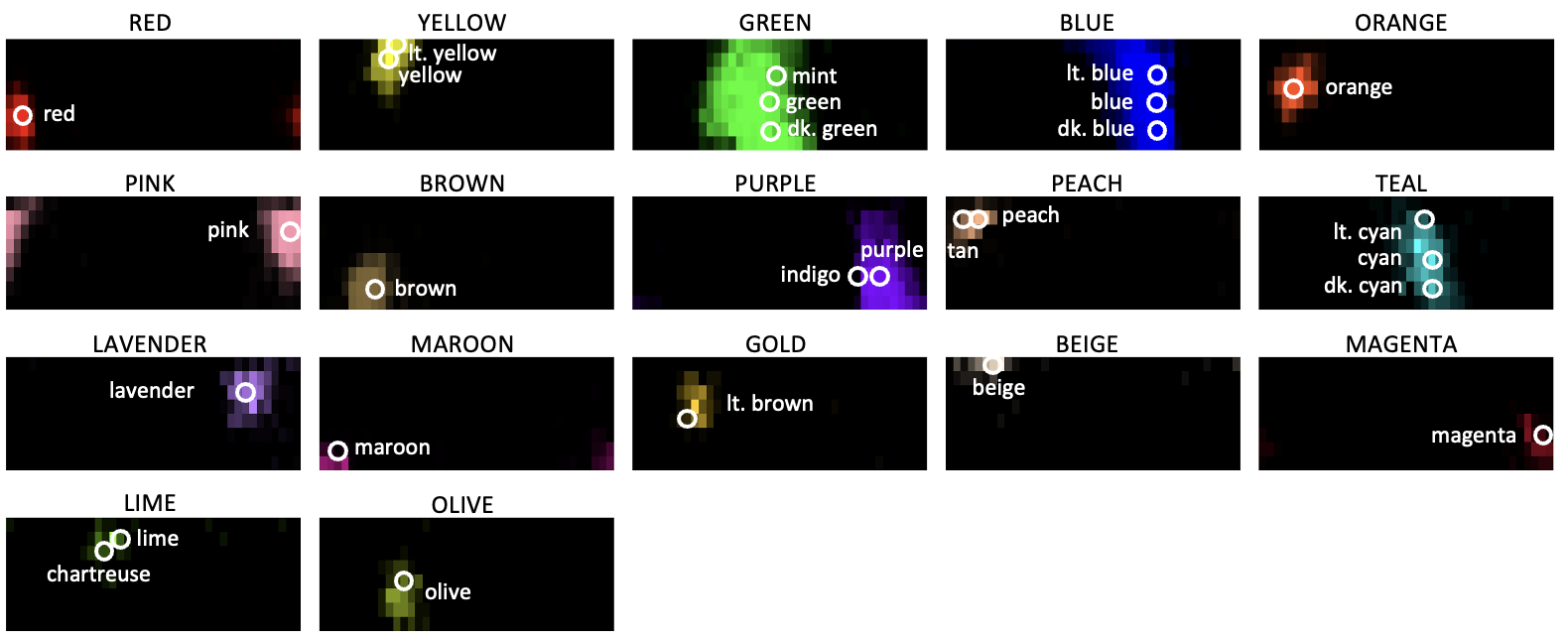


Figure S2. Relative frequency plots of the 17 English chromatic categories determined by cluster analysis of 51 speakers of American English from Lindsey & Brown [2]. Here “frequency” refers to the number of times a WCS color tile was included among all lexical color groups assigned by cluster analysis to that particular category. Format of each graph as in Fig. S1, except sidebar containing achromatic tiles omitted for clarity. Capitalized names: nominal category designations from Lindsey & Brown; lowercase names: our standard designations as in Fig. S1 and in main text.

The color palette also included good examples of the Somali lexical color categories. Figure S3 shows the same 30 samples mapped onto the eight principal color categories of the Somali lexicon [1]. Note that, with the exception of *GRUE*, these categories correspond nicely to many of the eight English basic chromatic color categories. However, while LCGs are localized to these regions, only LCGs falling into the GUDUUD (RED), CAGAAR (GREEN), and BULUUG (BLUE) regions of the color chart are named with high consensus (WHITE and BLACK are also high consensus, but are not shown in Fig. S2). These results agree with those of Maffi (1990), who concluded from her linguistic analysis that the Somali color lexicon lacks basic color terms for BROWN, PINK, ORANGE, and PURPLE, and GRAY.

Other characteristics of the Somali color lexicon of note are: 1) JALLE spans both the YELLOW and ORANGE English categories (c.f. Fig. 4 of main text). A few Somali informants used oranji to name some orangish colors in the JAALE group, and pinks and purples sometimes appeared in the JAALE group (barely visible in Fig. S3). 2) GUDUUD extends well beyond the range of English RED, including colors English speakers would label orange, pink, and/or purple. 3) Some Somali informants do not lexically distinguish between blues and greens and some purples, establishing a *GRUE* category [see Ref. 1 for more details]. These three characteristics were also common throughout the World Color Survey among languages thought to be in the early stages of color term evolution [see Refs. 3, 5, 6, for further discussion]. The palette was well-suited to reveal a term for *GRUE* if participants wished to use it (last panel in Fig. S3), but there is no evidence for this in Figs. 4A or 4D of the main text.


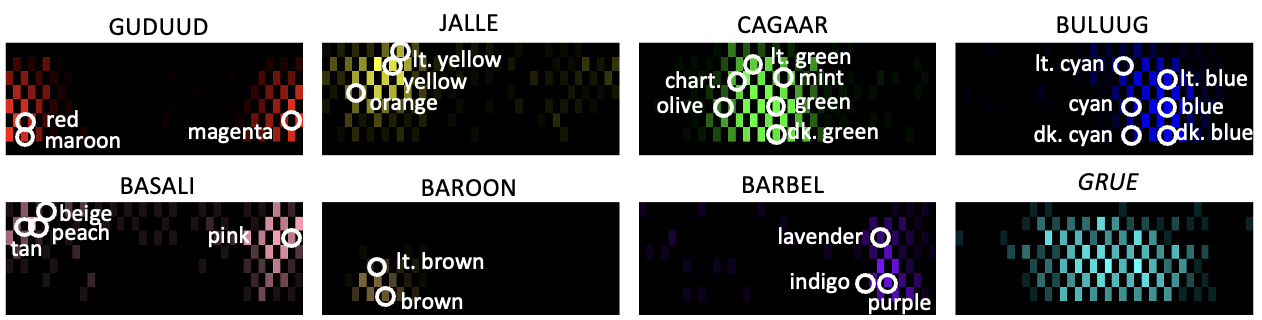


Figure S3. Relative frequency plots of the 8 principal Somali chromatic categories determined for 28 native speakers of Somali [from 1]. Capitalized names: modal Somali terms for each category, except *GRUE*, a commonly used portmanteau of “green or blue”; Otherwise, format the same as that in Fig. S2. See text for further details.

As a check on our palette design, we calculated MI for English and Somali using the color naming data from our previous studies [1, 2]. The new analysis paired all possible dyads of informants in those studies, and they included either all the data, or just the data from the 30-color subset of the WCS palette used here. The results of those analyses (Table S1) were similar to the dyadic MIs from the present study.

**TABLE S1**

Comparisons of dyadic Mutual Information based on past and present color-naming studies,

|  | Somali [1] | | |  | | English [1] | |
| --- | --- | --- | --- | --- | --- | --- | --- |
| Previous studies [2] | | 1.74 ± 0.02 |  | | 2.9± 0.02 | |  |
| 30-sample calculated [3] | | 1.59 ± 0.04 |  | | 2.81± 0.02 | |  |
| 30-sample observed naïve [4] | | 1.71 ± 0.11 |  | | 2.77± 0.10 | |  |
| 30-sample observed exp. [4] | | NA |  | | 2.90± 0.12 | |  |

[1] Means ± 95% confidence intervals

[2] Based on all color naming data from Brown, et al. [1] and Lindsey & Brown [2] respectively.

[3] Based on the present 30-sample subset of color naming data from [refs. 1, 2]

[4] Results from dyadic color naming data from present study

The stimuli and lighting environments used here were quite different from those used in color naming experiments involving Munsell tiles. Our previous English and Somali color naming studies used physical tiles from the Munsell Book of Color, which were viewed in a lightbox under _~_5000K illuminant, whereas here the Color Communication Game was played in nominal room illumination using samples displayed on iPads with a white point of 6500K. To compensate for the differences between these two viewing environments, lab personnel made perceptual matches of the iPad stimuli to the corresponding Munsell papers (in the lightbox) using an asymmetric color matching procedure. Thus, the test palette colors shown in Figs. S1—S3 are associated with the Munsell samples based on careful perceptual matches, but not necessarily on chromaticity matches. Table S2 shows the chromaticities of our stimuli, calibrated using the PR650 SpectraScan® spectral photometer (PhotoResearch, Chatsworth, CA).

**TABLE S2**

*Specifications of the 30 colors in the Color Communication Game test palette*

| # | Color [1] | Munsell [2] | WCS [3] | x [4] | y | Y |
| --- | --- | --- | --- | --- | --- | --- |
| 1 | magenta | 5RP 4/10 | G39 | 0.461 | 0.229 | 8.55 |
| 2 | pink | 7.5RP 7/8 | D39 | 0.378 | 0.29 | 37.5 |
| 3 | maroon | 7.5R 3/8 | I03 | 0.604 | 0.352 | 2.89 |
| 4 | red | 7.5R 4/12 | G03 | 0.629 | 0.336 | 8.6 |
| 5 | tan | 7.5R 8/6 | C03 | 0.408 | 0.347 | 54.2 |
| 6 | peach | 5YR 8/8 | C05 | 0.426 | 0.381 | 54.8 |
| 7 | orange | 5YR 6/12 | E06 | 0.543 | 0.409 | 37.4 |
| 8 | beige | 5YR 9/2 | B06 | 0.373 | 0.382 | 80.7 |
| 9 | lt. brown | 2.5Y 5/8 | F08 | 0.468 | 0.466 | 14.6 |
| 10 | brown | 2.5Y 3/4 | H09 | 0.48 | 0.452 | 4.1 |
| 11 | yellow | 5Y 8/12 | C10 | 0.451 | 0.479 | 68.8 |
| 12 | lt. yellow | 7.5Y 9/6 | B11 | 0.406 | 0.456 | 88 |
| 13 | olive | 10Y 5/6 | G12 | 0.427 | 0.51 | 17.2 |
| 14 | chartreuse | 5GY7/10 | C14 | 0.373 | 0.476 | 45.9 |
| 15 | lime | 10GY 8/8 | C16 | 0.332 | 0.483 | 57.7 |
| 16 | dk. green | 7.5G 3/8 | H19 | 0.29 | 0.552 | 5.41 |
| 17 | green | 7.5G 5/8 | F19 | 0.26 | 0.443 | 11 |
| 18 | mint | 2.5BG 7/8 | D20 | 0.257 | 0.42 | 59.6 |
| 19 | lt. cyan | 7.5BG 8/6 | C23 | 0.24 | 0.345 | 57.7 |
| 20 | dk. cyan | 2.5B 3/6 | H24 | 0.215 | 0.294 | 8.11 |
| 21 | cyan | 7.5BG 5/8 | F24 | 0.23 | 0.336 | 24.8 |
| 22 | dk. blue | 2.5PB 3/10 | H29 | 0.181 | 0.174 | 5.14 |
| 23 | blue | 2.5PB 5/12 | F29 | 0.195 | 0.217 | 15.6 |
| 24 | lt. blue | 2.5PB 7/8 | D29 | 0.251 | 0.271 | 37.2 |
| 25 | indigo | 7.5PB4/12 | G31 | 0.22 | 0.189 | 11.4 |
| 26 | lavender | 2.5P 7/6 | D33 | 0.276 | 0.244 | 40.3 |
| 27 | purple | 5P 4/10 | G34 | 0.266 | 0.156 | 9.53 |
| 28 | black | N0.5/0 | J00 | 0.317 | 0.332 | 0 |
| 29 | gray | N5.25/0 | E00 | 0.319 | 0.336 | 23.8 |
| 30 | white | N9.5/0 | A00 | 0.316 | 0.332 | 99.6 |

____________________________

[1] Names used to reference color samples used in this section and in the main text.

[2] Munsell chip closest to corresponding iPad color, as determined by asymmetric color matching.

[3] World Color Survey coordinates. A-J: rows, top to bottom; 1 – 40: columns left to right.

[4] x, y and Y are CIE chromaticity coordinates and luminance (in cd/m^2^) of each iPad test color.

2. **Instruction scripts**

**2.1 Somali instruction script (presented by the interpreter)**

At the beginning of the sender phase:

*“You will participate in the study using this iPad computer.*

*You will see some colored squares, one at a time.*

*Please tell me the name of that color, using a single word.*

*The researcher will enter it into the computer for you.*

*There are no “right” or “wrong” answers, as long as you use only a single word for each color. You may also say ‘I don’t know’ if you don’t know a word for a color.”*

At the beginning of the receiver phase:

*“Now, you will see some color names on the computer, one at a time, which I (the interpreter) will read to you.*

*These names were provided by you or by another person.*

*And below the color names you will see some colors.*

*Please touch the color that you think you or the other person was viewing at the time that you or they told us the color name.”*

**2.4 English instruction scripts**

The naïve run.

At the beginning of the sender phase:

*“You will participate in the study using the iPad computer at your place at the table.*

*On each trial, you will see a colored square.*

*Please enter a one-word name of that color into the space provided, using the keyboard shown on the iPad screen.*

*There are no “right” or “wrong” answers, as long as you use only a single word for each color. You may also enter ‘DK’ for ‘I don’t know’ if you don’t know a word a color.*

*The baffle is to prevent you from seeing other peoples’ iPads… please respect it!”*

At the beginning of the receiver phase:

*“Now, you will see some color names on the computer, one at a time.*

*These names were provided by you or by another person.*

*And below the color names you will see some colors.*

*Please touch the color that you think you or the other person was viewing at the time that you or they told us the color name.*

*Again, please respect the baffle.”*

The experienced run.

At the beginning of the sender phase:

*“So, now that you understand the game, we will play it again.*

*Once again, please enter a one-word name for each color, which you or another player will use later to identify the color you were viewing when you provided the name. You may enter ‘DK’ for ‘I don’t know’ if you don’t know a word for a color.*

*Again, a single word for each color, and please respect the baffle.”*

At the beginning of the receiver phase:

*“Just like before, you will see the color names that were by you or by another person in the group.*

*And below the color names you will see the colors.*

*Please touch the color that you think you or the other person was viewing at the time that you or they told us the color name.”*

Closing comment:

*“Thank you for participating. Now, please do not tell others about the procedures in this study: it is important that the first round of the game be played by completely naïve participants, so we don’t want rumors to spread to the likely participant pool.”*

**3. Table of results from Figs. 3 and 6.**

|  | Mutual Information (bits) | | | | | | | | |
| --- | --- | --- | --- | --- | --- | --- | --- | --- | --- |
|  | self | | | |  | dyads | | | |
|  | N |  | mean | SD |  | N |  | mean | SD |
| Somali | 63 |  | 3.069 | 3.043 |  | 63 |  | 1.708 | 0.448 |
| English naïve | 31 |  | 4.188 | 0.404 |  | 70 |  | 2.769 | 0.419 |
| English exp | 31 |  | 4.588 | 0.228 |  | 70 |  | 2.896 | 0.511 |
|  |  |  |  |  |  |  |  |  |  |
|  |  |  |  |  |  |  |  |  |  |
|  | Simulated Exactly-Correct (bits) | | | | | | | | |
|  | self | | | |  | dyads | | | |
|  | N |  | mean | SD |  | N |  | mean | SD |
| Somali | 63 |  | 3.393 | 0.274 |  | 63 |  | 1.627 | 0.453 |
| English naïve | 31 |  | 4.357 | 0.315 |  | 70 |  | 3.443 | 0.201 |
| English exp | 31 |  | 4.663 | 0.171 |  | 70 |  | 3.720 | 0.297 |
|  |  |  |  |  |  |  |  |  |  |
|  |  |  |  |  |  |  |  |  |  |
|  | Exactly-Correct (bits) | | | | | | | | |
|  | self | | | |  | dyads | | | |
|  | N |  | mean | SD |  | N |  | mean | SD |
| Somali | 63 |  | 2.801 | 0.532 |  | 63 |  | 2.529 | 0.487 |
| English naïve | 31 |  | 4.073 | 0.339 |  | 70 |  | 3.916 | 0.201 |
| English exp | 31 |  | 4.458 | 0.195 |  | 70 |  | 4.136 | 0.231 |
|  |  |  |  |  |  |  |  |  |  |
|  |  |  |  |  |  |  |  |  |  |
|  | Categorically-Correct (bits) | | | | | | | | |
|  | self | | | |  | dyads | | | |
|  | N |  | mean | SD |  | N |  | mean | SD |
| Somali | 63 |  | 4.524 | 0.324 |  | 63 |  | 3.458 | 0.590 |
| English naïve | 31 |  | 4.779 | 0.101 |  | 70 |  | 3.944 | 0.094 |
| English exp | 31 |  | 4.774 | 0.077 |  | 70 |  | 3.876 | 0.130 |
|  |  |  |  |  |  |  |  |  |  |
|  |  |  |  |  |  |  |  |  |  |
|  | Terms Per Receiver (bits) | | | | | | | | |
|  | self | | | |  | dyads | | | |
|  | N |  | mean | SD |  | N |  | mean | SD |
| Somali | 63 |  | 3.354 | 0.273 |  | 63 |  | 3.354 | 0.273 |
| English naïve | 31 |  | 4.354 | 0.326 |  | 70 |  | 4.368 | 0.313 |
| English exp | 31 |  | 4.662 | 0.171 |  | 70 |  | 4.664 | 0.170 |

**4. Bar graphs from choice analysis.**

Although some color terms covered a large domain of color samples, receivers speaking both languages often chose only a narrow range of samples as their responses (see Fig. 4 of the main text). Figure S4 shows the distributions of the color terms for the first 6 Basic Color Terms of Berlin & Kay (panels A—C) and the distributions of the corresponding choices (panels D—F). The choices were generally located near the centers of the domains of the color terms. This trend was less pronounced in the experienced English data set because the color terms generally covered narrower ranges of samples. These results suggest an association between the chosen samples and the focal colors of Berlin & Kay.

Fig. S4. Distributions of the color terms and color choices for selected color categories, coded by the colors of the bars. A—C, color-naming data sent as “messages,” pooled across synonymous terms. D—F, color sam­ples selected by the receivers in response to these messages. The data set for each color term was normalized to its maximum. The distributions of the choice data were narrower than the distributions of the naming data whenever the naming data were wider than two samples.

REFERENCES

1. Brown, A.M., Isse, A., and Lindsey, D.T. (2016). The color lexicon of the Somali language. Journal of Vision *16*.

2. Lindsey, D.T., and Brown, A.M. (2014). The Color Lexicon of American English. Journal of Vision *14*, 17.

3. Lindsey, D.T., and Brown, A.M. (2006). Universality of color names. Proceedings of the National Academy of Sciences of the United States of America *103*, 16608-16613.

4. Berlin, B., and Kay, P. (1969). Basic Color Terms: Their Universality and Evolution, (Berkeley and Los Angeles: University of California Press).

5. Lindsey, D.T., and Brown, A.M. (2009). World color survey color naming reveals universal motifs and their within-language diversity. Proceedings of the National Academy of Sciences of the United States of America *206*, 19785 -- 19790.

6. Lindsey, D.T., Brown, A.M., and Lange, R. (2020). Testing the Cross-Cultural Generality of Hering’s Theory of Color Appearance. Cognitive Science *44*, e12907.
